# Commentary: Time trends in the incidence of essential tremor: Evidences from UK and France primary care data

**DOI:** 10.3389/fneur.2023.1136150

**Published:** 2023-01-20

**Authors:** Elan D. Louis

**Affiliations:** Department of Neurology, University of Texas Southwestern Medical Center, Dallas, TX, United States

**Keywords:** essential tremor, incidence, epidemiology, population-based, disease burden

## Introduction

I read the paper by Antonazzo et al. on time trends in the incidence of essential tremor (ET) with great interest (1). The authors should be congratulated for contributing to the scant published literature in this area. As the authors noted, despite the extraordinarily high prevalence of ET (2, 3), prior to their report (1), there had been a mere two studies of the incidence of ET (4, 5). One of these was in Spain (4) and the other was in the United States (5). As noted by Antonazzo et al. (1), such studies are of value in estimating the number of newly emerging cases and planning health care resources. In their study, the authors aimed to evaluate the incidence of ET in the United Kingdom (UK) and France, between the years 2013 and 2020, using two large independent primary care databases (1).

The authors identified 4,970 and 4,905 incident ET cases in the UK and France, respectively. The yearly average standardized incidence rate (per 100,000 person-Years [py]) was 19.51 (the UK) and 19.50 (France). Analyses showed a significant increase in incidence with age, higher incidence among males, and slightly increasing trends in incidence in both countries.

## Comment on several items

Here I take the opportunity to comment on several items that deserve additional attention. First, as the authors note, the database that they used was intended for the purposes of patient management rather than medical research (1). Stated in another way, the study used a practitioner database rather than employing a population-based design. There is a wealth of data, from studies across numerous countries, showing that the proportion of ET cases in the population who are diagnosed by physicians is between 7.2 and 12.3% (6). Hence, ~90% of ET cases dwelling in the population have not been recognized by their treating physicians and the diagnosis has not been assigned to them. As a result, studies that use treatment-based databases are ascertaining a small percentage of the total disease burden. The authors write that “ET is a chronic degenerative disease that requires continuous care, therefore it is implausible that this diagnosis has never been reported in a symptomatic patient's records.” Despite being both chronic and progressive, most patients with ET do not seek medical attention for their tremor, as noted above. Furthermore, an individual may see a health care provider for another primary reason, such as hypertension or diabetes, and during the clinical encounter, they may mention to the provider that tremor is an additional issue of concern. Even that is rare. In a population-based study in Finland in which 171 ET cases were identified, 19 (11.1%) had sought medical advice for tremor prior to their study examination, and an additional 14 (8.2%) had mentioned tremor to a physician while seeking help for some other reason (7). All this is to say that databases from health care settings will greatly underestimate the true disease burden. It is for this reason that the present study (1), as well as that of Rajput et al. (5), which similarly used medical records to ascertain ET cases, and which reported an incidence of 18.3/100,000 py in males and 17.1/100,000 py in females, provided estimates of ET incidence that were low (1). In the Rajput study (5), the incidence rate for ET peaked in those age 80+ years, where it was 84.3/100,000 py. In the present study, this value was 49.27/100,000 py (the UK) and 51.52/100,000 py (France) in the same age group (1). By contrast, in the Benito-Leon study (4), which was population-based, the adjusted annual incidence rate in the population aged ≥65 years was 616/100,000 py, a value >10 times that of these two treatment-based studies. This difference across studies fits with the data that approximately 90% of ET cases are not ascertained in treatment-based databases.

Second, the authors raised a concern about the Benito-Leon study (4). They wrote: “Indeed, participation in the Spanish study…was voluntary and individuals might have been more prone to participate if they already had some neurological symptoms. Therefore, this selective recruitment might have contributed to overestimating the observed phenomenon (1).” The Spanish study had two phases: Phase 1, in which the prevalence of ET was estimated, and Phase 2, in which incidence of ET was estimated (4). During Phase 2, 367 declined participation. By comparison, the final Phase 2 cohort comprised 3,942 participants. Even if we were to assume that none of the 367 who declined had had ET, the effect on the estimate of incidence would have been low (i.e., likely less than a 10% reduction). Furthermore, as noted in the paper, the incidence estimate was likely low for several reasons—the use of a screening questionnaire and the fact that nine who screened positive were not included in the final cohort (4).

The authors found an age-associated increase in the incidence of ET, which has been noted in prior studies (4, 5). They also reported a higher incidence in males. This is an intriguing finding, although one that is a challenge to interpret, as the prevalence of ET is similar in males and females (3) and there has been no observed sex-associated survival advantage in ET (8).

They also reported a slightly increased incidence in ET throughout the study period. This could have been the result of an increase in recognition of ET during that period. If it reflected a true increase in disease incidence, it would be of great value to learn more.

## Summary

These authors should be lauded for estimating incidence of ET in a primary care setting. As I point out, the population-based estimate of disease incidence is substantially higher (4). Regardless, disease burden is high in both settings and data from both settings are of high value. As the world's population ages, the burden of ET is likely to rise over time. Given the disability associated with ET, and the observation that it is a risk factor for Parkinson's disease (9) and dementia (10), attempts at disease prevention would be highly worthwhile.

## Author contributions

The author confirms being the sole contributor of this work and has approved it for publication.
